# Buffalo Academy of Medicine (Constitution and By-Laws)

**Published:** 1892-07

**Authors:** 


### BUFFALO ACADEMY OF MEDICINE.

### Constitution.

#### ARTICLE I.—NAME.

This association shall be called "The Buffalo Academy of Medicine," and shall be composed of resident, non-resident, and honorary fellows, and benefactors.

#### ARTICLE II.—OBJECT.

The object of the Academy shall be the promotion of the science and art of medicine, including the maintenance of a medical library and a museum.

### ARTICLE III.—MEMBERS.

SECTION 1.—The membership of the Academy shall be composed of the present active members, in good standing, of the Buffalo Medical and Surgical Association, the Buffalo Obstetrical Society, the Buffalo Pathological Society, and the Buffalo Clinical Society.

SEC. 2.—Hereafter each candidate for resident fellowship must have been a graduate in medicine, in active practice, for the two years last preceding the application, and must be a resident of the City of Buffalo.

SEC. 3.—Resident fellows removing from the city may become non-resident fellows by vote of the council; upon resuming residence in the city they may be re-admitted to resident fellowship in the same way.

SEC. 4.—Surgeons and medical officers of the United States Army, Navy, or Marine Hospital Service, and physicians residing outside the City of Buffalo, may be admitted as non-resident fellows, and on becoming residents of the city they may, on payment of the difference in initiation fees and by vote of the council, be elected resident fellows.

SEC. 5.—Honorary fellows must be men of eminence, distinguished in science. They shall be chosen by vote of the Academy upon recommendation of the council. The number of honorary fellows shall be limited to twenty-five.

SEC. 6.—Any person having rendered illustrious service to the Academy may be elected a benefactor, by vote of the Academy, upon recommendation of the Council.

### ARTICLE IV.—OFFICERS.

SECTION 1.—The officers of the Academy shall consist of a president, four vice-presidents, a secretary, a treasurer, three trustees, and a board of councillors, which shall consist of the aforementioned officers and the secretaries of the various sections.

SEC. 2.—The President shall be elected by the Academy, by ballot, for the term of one year.

SEC. 3.—The vice-presidents shall be the chairmen of the sections on medicine, surgery, obstetrics, and pathology; they shall be elected as each section may designate.

SEC. 4.—The Secretary shall be elected by the Academy, by ballot, for the term of two years.

SEC. 5.—The treasurer shall be elected by the Academy, by ballot, for the term of one year.

SEC. 6.—The trustees shall be elected by the Academy, by ballot, for a term of three years, one being elected and one retiring each year. At the first meeting of the trustees they shall draw lots as to their respective terms of office.

### ARTICLE V.—SECTIONS.

SECTION 1.—The Academy shall be divided into sections as follows: A section on general medicine (including materia medica and therapeutics), now known as The Buffalo Clinical Society; a section on surgery (including ophthalmology, otology, laryngology, genito-urinary and orthopedic surgery), now known as The Buffalo Medical and Surgical Association; a section on obstetrics and gynecology, now known as The Buffalo Obstetrical Society; a section on anatomy, physiology, and pathology, now known as The Buffalo Pathological Society; and as many more as may at any time be proposed by ten resident fellows of the Academy, and be approved by vote of three-fourths of the resident fellows present at a stated meeting.

SEC. 2.—Each resident or non-resident fellow at the time of his election, shall designate to the president of the Academy what section or sections he wishes to join, and shall be assigned, by the president, to such section or sections, and shall become a member of the same upon complying with the requisitions for membership in such section or sections.

SEC. 3.—Each section shall prescribe its own requirements for admission and other regulations for its government, provided that none of them is antagonistic or contrary to the regulations of the Academy.

### ARTICLE VI.—DISCIPLINE.

SEC. 1.—The Academy may expel or suspend a fellow for violation of its regulations.

SEC. 2.—A three-fourths vote of the resident fellows present shall be necessary to expel or suspend a fellow. This vote shall not be taken unless the printed call for the meeting contains a notice to suspend or expel a fellow.

ARTICLE VII.

Each fellow of the Academy shall be furnished with a copy of the Constitution and By-laws.

ARTICLE VIII.—AMENDMENTS.

No part of the Constitution shall be altered or amended except by a vote of three-fourths of the resident fellows present at a stated meeting, subsequent to one at which a proposition to that effect shall have been made in writing. Notice to this effect shall be included in the call for this meeting.

---

### By-Laws.

### I.

MEETINGS.

Section 1.—The stated meetings of the Academy shall be held quarterly, in the months of September, December, March, and June, on such day as the council may determine.

Sec. 2.—The annual meeting, for the election of officers and the reading of annual reports, shall be held in June, one week after the stated meeting in June.

Sec. 3.—Special meetings may be called at any time by the president, and shall be called upon the requisition, in writing, of ten of the resident fellows.

Sec. 4.—When practicable, three days' notice shall be given for special meetings, and no business shall be transacted at such meetings except that stated in the call.

### II.

QUORUM.

At all meetings fifteen resident fellows shall constitute a quorum.

### III.

ELECTION.

At the stated meeting in March, all nominations for the officers to be elected by the Academy shall be made, to be voted for by ballot, between the hours of 4.00 and 6.00 p. m., on the day of the annual meeting in June. The candidate receiving the highest number of votes cast shall be declared elected. No fellow whose annual dues remain unpaid shall be entitled to vote.

### IV.

### REPORTS.

At the annual meeting in June, a report shall be presented by the council, by the secretary, by the treasurer, by the trustees, and by each section on the work it has done during the year, the annual address of the retiring president shall be delivered, and the newly elected officers shall be duly installed.

### V.

### BUSINESS.

The sections shall bring before the Academy at its stated meetings, or as otherwise ordered, such matters as pertain to the several departments respectively assigned to them, as subjects for discussion or other action. They shall severally examine and report on all papers, documents, instruments, etc., submitted to them, and give a brief abstract of the proceedings of the section. They shall each furnish a paper, or prepare a discussion for a stated meeting of the Academy once a year.

### VI.

### THE PRESIDING OFFICERS.

The president shall preside over all meetings, appoint all committees not otherwise provided for, announce the results of all votes, and be, ex-officio, a member of all standing committees.

### VII.

Section 1.—The vice-presidents shall assist the president in the discharge of his duties. At any stated meeting, in the absence of the president, that vice-president shall preside who is chairman of the section presenting the programme of the evening.

Sec. 2.—At the annual meeting, or at any special meeting, in the absence of the president, the vice-presidents shall preside in the following order: Chairman of section on medicine, chairman of section on surgery, chairman of section on obstetrics, chairman of section on pathology.

### VIII.

### SECRETARY.

Section 1.—The secretary shall keep minutes of the proceedings of all meetings, issue notices of meetings, notify officers and members of committees of their appointment, transact such other business as may be ordered by the Academy or council, and shall be, ex-officio, a member of all appointive committees.

### IX.

### TREASURER.

Section 1.—The treasurer shall receive the initiation fees and signatures of newly admitted fellows, receive all dues and assessments, keep a correct account of all money received and expended, give a statement of all funds of the Academy in his care to the council or the Academy annually, and whenever requested by either.

Sec. 2.—He shall report to the council the names of all fellows who are one year, or more, in arrears for dues.

### X.

### TRUSTEES.

The trustees shall have charge of all real estate which is now in, or may at any time come into the possession of the Academy. It shall be their duty to invest the funds of the Academy, report the investment and condition of the funds and property aforesaid to the council when required, and annually to the Academy. All funds and property in their care shall only be used as specified by the donors. All checks and all orders withdrawing deposits from any bank, trust company, or money institution, shall be signed by the chairman of the trustees.

### XI.

### COUNCIL.

The council shall supervise the general affairs and interests of the Academy, and provide suitable accommodations for its meetings and its movable property, and for its business transactions. It shall estimate for any annual assessment, and shall annually audit the financial account. It shall appoint the librarian and the custodian of the museum. A majority of the council shall constitute a quorum.

### XII.

### NEW MEMBERS.

Each candidate for resident or non-resident fellowship shall sign an application in writing, endorsed by two resident fellows personally acquainted with him. He shall be elected, by ballot, by the Academy upon recommendation by the council. An affirmative vote of three-fourths of the resident fellows present at a stated meeting shall be necessary to elect.

### XIII.

### FINANCE.

Section 1.—Each resident fellow, upon admission, shall pay an initiation fee of $10.00, which shall include the first year's annual dues; each non-resident fellow, an initiation fee of $3.00. These moneys shall go to the building fund, the principal, or interest, of which may be used for the construction or maintenance of the Academy.

Sec. 2.—The annual dues of each resident and each non-resident fellow shall be $5.00, payable in the month of September.

Sec. 3.—Any fellow failing to pay his dues for one year or more, shall be liable to forfeit his fellowship.

### XIV.

### MISCELLANEOUS.

Every duly elected resident fellow shall sign the constitution and by-laws within two months after election, and in default thereof, such election shall be deemed void, unless a reason satisfactory to the council shall be given.

### XV.

No one elected a resident or non-resident fellow shall be entitled to the rights and privileges of fellowship until the initiation fee shall have been paid, and the constitution and by-laws signed.

### XVI.

A copy of every memoir, discourse, or paper, read before the Academy shall be kept in its archives by the librarian.

### XVII.

A volume of transactions shall be published under the supervision of the council, when ordered by the Academy.

### XVIII.

Section 1.—The following shall be the order of business at the annual meeting :

1. Reading of the minutes of last annual meeting.
2. Reading of annual reports of council, of secretary, of treasurer, and of trustees.
3. Report of sections in their order : Medicine, surgery, obstetrics, pathology.
4. Address of retiring president.
5. Installation of newly elected officers.
6. Adjournment.

Sec. 2.—The following shall be the order of business at the stated meetings :

1. Reading of minutes of last stated meeting, and of all special meetings held since the last stated meeting.
2. At March meeting, nomination of officers.
3. Propositions for fellowship.
4. Admission of new fellows.
5. Reading of papers and discourses.
6. Appointed debates and discussions.
7. Unfinished business.
8. New business.
9. Adjournment.

### XIX.

No fellow shall have the privilege of speaking more than once on any question under consideration, except by permission of the Academy.

### XX.

### AMENDMENTS.

These by-laws may be suspended by unanimous vote at a stated meeting ; they may be repealed or amended by a three-fourths vote of the resident fellows present at a stated meeting, provided that notice of the same has been given, in writing, at a previous stated meeting, and has been announced on the call for the meeting when the vote is to take place.

### XXI.

### RULES.

Robert's "Rules of Order" shall be accepted as a parliamentary guide in the deliberations of this Academy.

---

Adopted at a joint meeting of the Buffalo Medical and Surgical Association, Buffalo Obstetrical Society, Buffalo Pathological Society, and Buffalo Clinical Society, held May 17, 1892.

WM. C. KRAUSS, M. D.,
*Secretary.*

DELANCEY ROCHESTER, M. D.,
*Chairman.*

